# Intersexual Differences in the Gene Expression of *Phoneutria depilata* (Araneae, Ctenidae) Toxins Revealed by Venom Gland Transcriptome Analyses

**DOI:** 10.3390/toxins15070429

**Published:** 2023-06-30

**Authors:** Diego Sierra Ramírez, Juan F. Alzate, Yuri Simone, Arie van der Meijden, Giovany Guevara, Lida Marcela Franco Pérez, Julio César González-Gómez, Carlos F. Prada Quiroga

**Affiliations:** 1 Grupo de Investigación Biología y Ecología de Artrópodos (BEA), Facultad de Ciencias, Universidad del Tolima, Altos de Santa Helena, Ibagué 730001, Colombia; dsierrar@ut.edu.co (D.S.R.); jcesargonzalez@ut.edu.co (J.C.G.-G.); 2 Centro Nacional de Secuenciación Genómica (CNSG), Facultad de Medicina, Universidad de Antioquia, Medellín 050010, Colombia; 3 CIBIO/InBIO/Biopolis, Campus Agrário de Vairão, Rua Padre Armando Quintas 7, 4485-661 Vila do Conde, Portugal; yurisimone1@gmail.com (Y.S.); mail@arievandermeijden.nl (A.v.d.M.); 4 Grupo de Investigación en Zoología (GIZ), Facultad de Ciencias, Universidad del Tolima, Altos de Santa Helena, Ibagué 730001, Colombia; gguevara@ut.edu.co; 5 Facultad de Ciencias Naturales y Matemáticas, Universidad de Ibagué, Carrera 22 Calle 67, Ibagué 730001, Colombia; lida.franco@unibague.edu.co

**Keywords:** next-generation sequencing, spider, transcriptomics, venomics, venom gland

## Abstract

The wandering spider, *Phoneutria depilata*, is one of Colombia’s most active nocturnal arthropod predators of vertebrates and invertebrates. Its venom has been a relevant subject of study in the last two decades. However, the scarcity of transcriptomic data for the species limits our knowledge of the distinct components present in its venom for linking the mainly neurotoxic effects of the spider venom to a particular molecular target. The transcriptome of the *P. depilata* venom gland was analyzed to understand the effect of different diets or sex and the impact of these variables on the composition of the venom. We sequenced venom glands obtained from ten males and ten females from three diet treatments: (i) invertebrate: *Tenebrio molitor*, (ii) vertebrate: *Hemidactylus frenatus*, and (iii) mixed (*T. molitor* + *H. frenatus*). Of 17,354 assembled transcripts from all samples, 65 transcripts relating to venom production differed between males and females. Among them, 36 were classified as neurotoxins, 14 as serine endopeptidases, 11 as other proteins related to venom production, three as metalloprotease toxins, and one as a venom potentiator. There were no differences in transcripts across the analyzed diets, but when considering the effect of diets on differences between the sexes, 59 transcripts were differentially expressed. Our findings provide essential information on toxins differentially expressed that can be related to sex and the plasticity of the diet of *P. depilata* and thus can be used as a reference for venomics of other wandering spider species.

## 1. Introduction

Animal venoms are cocktails of bioactive compounds that alter the normal physiology of the envenomed animal, delivered into the victim through specialized body structures like stingers or fangs [1,2,3]. Venom is a multifunctional trait, mainly used in three different essential ecological contexts: prey subjugation (e.g., in spiders, snakes, scorpions, parasitoid wasps, cnidarians, coleoids, and centipedes), defense against attackers (e.g., in snakes, bees, ants, some species of amphibians, and fishes), and sexual-related contexts (e.g., in the platypus and scorpions) [1,3,4,5]. This biochemical weapon represents an evolutionary innovation and ecological adaptation that has evolved independently in more than 100 phylogenetically divergent lineages of the metazoa [2,6,7]. Within the arthropods, spiders are among the most abundant terrestrial predators. They are a megadiverse group with about 50,000 extant species and new species continually being discovered [8,9]. They play an important role in controlling prey populations in most terrestrial ecosystems [10,11,12]. Although most spiders prefer to prey on a wide variety of arthropods, including other spiders [11,13], ten spider families (Araneidae, Atracidae, Ctenidae, Lycosidae, Nephilidae, Pisauridae, Theraphosidae, Theridiidae, Trechaleidae, and Sparassidae) are habitual vertebrate-eaters or do so to supplement their arthropod diet [14,15,16,17]. The evolution of hunting techniques in spiders has been closely linked to their different foraging niches, ranging from aerial webs to complex hunting strategies in wandering spiders [9,13]. However, the predatory success of spiders can be summarized in two main characteristics: silk and venom production. These two innovations are used synergically or exclusively and allow prey manipulation and subjugation [9,18]. Additionally, all spiders are liquid feeders, and the cytolytic compounds of venom may facilitate the pre-digestion of the prey required for feeding [9].

Given the distinct ecological roles of spider venom in predation, defense, and digestion, it is not surprising that it also has an unprecedented degree of chemical complexity, containing up to 3000 different molecules [9,19]. Studies on other venomous organisms, such as snakes, found evidence that diet breadth is an important factor in venom complexity [20,21]. Spider venom has been extensively investigated but in a very limited number of medically significant species. Although the ecological differences in predatory contexts have been often overlooked, this topic saw an increased interest in the last five years due to the pharmaceutical potential and agricultural applications of spider venom peptides [22,23]. Earlier studies have estimated that the venom of a spider contains around 200 toxins [24]. However, there is evidence that different environmental and biological factors, such as geography [25,26], season [27], ontogeny [28,29,30], sex [31,32,33], and diet [31,34,35] affect venom composition [9]. In addition, this high variability could be related to the long-term predator–prey coevolution process [20]. For example, recent studies on the composition of the venom of spiders of the genus *Latrodectus* suggest that the presence of molecules such as α-latrotoxin, a molecule acting specifically on the nervous system of vertebrates, could be related to the consumption of small reptiles and amphibians that are occasionally included in the diet of these spiders [36]. Conversely, the development of this strong toxin in the genus *Latrodectus* has been proposed as a defense mechanism against predators [37]. There are examples of spider species specialized in certain prey (e.g., myrmecophagy), in which the venom composition is simpler in comparison with generalist spiders, but the few toxins have a high affinity for and effectiveness upon their target prey [38].

The spider genus *Phoneutria* (family Ctenidae) currently comprises nine large (17–48 mm) species, popularly known as “banana spiders.” They are distributed from Costa Rica to northern Argentina [39,40]. These wandering and nocturnal spiders have medically important venom with mainly neurotoxic action [41,42]. The risks to human health are aggravated by their aggressive defensive behavior and synanthropic habits [41,43]. Due to its high clinical significance in Brazil, the venom of *Phoneutria nigriventer* is the most studied and is often used as reference across the other species of *Phoneutria* [33]. Nevertheless, in the past few years, venoms from different species of the genus *Phoneutria* have started to gain more attention and in more ecological contexts rather than their mere application in medical or agricultural practice.

*Phoneutria depilata* is a widespread species distributed from Central America to the Trans-Andean region of Colombia and Ecuador. It is mainly found in both dry and humid tropical forests (0–1700 m a.s.l.), usually on the ground on sparse litter or on low vegetation [39,41]. Valenzuela-Rojas et al. [43] studied the venom of *Phoneutria depilata* (as *P. boliviensis* see [39]), revealing that despite no intersexual difference in prey selection, males produced a smaller quantity of (equally lethal) venom than females. A metabarcoding analysis of gut content in this species has shown a wide range of prey, including cockroaches, crickets, small insects, other spiders, and small vertebrates [44]. In addition, intersex and interpopulation differences in prey were also observed.

Through transcriptome analysis, researchers can determine the expression of components associated with venom from just a few samples [45]. A venom gland transcriptomic analysis may be employed as an alternative to determine the sequences of cDNA that encode specific peptide toxins including the identification of new genes, as well as differential gene expression analysis [45]. The transcriptome analysis of the venom glands of *Phoneutria pertyi* revealed 296 unique toxin sequences [46]. A recent transcriptomic study detected a total of 682 transcripts that were identified as potentially coding for venom component toxin sequences from the venom glands of *P. depilata* [47]. Most of the transcripts found were neurotoxins (156), but other transcripts coded for enzymes (239), growth factors (48), clotting factors (6), and a diuretic hormone (1). However, this study only identified potential peptides and proteins in the venom from a single individual. Due to the wide range of prey species preyed upon by *P. depilata*, a transcriptome analysis of the gland venom of this species may provide a good model to study selective toxin expression across sex and/or diet.

In other arachnids, venom composition variation has been linked to environmental variables, such as perceived predation risk and diet composition [48]. In addition, intersex differences in venom composition have been well established in arachnids [49]. Since intersex and interpopulation differences in prey preference exist in *P. depilata* [44], we propose that these two factors may influence venom composition, as shown by toxin gene expression. In this work, we therefore test for: (I) differential expression of venom gland genes between sexes and (II) a transcriptional response in the venom gland of *P. depilata* in response to diet. To test the effect of these two factors on gene expression, we performed a comparative analysis to identify differential gene expression through de novo transcriptome sequencing technique in twenty adult specimens of *P. depilata*, sorted according to males and females and between dietary differential treatments.

## 2. Results

### 2.1. Transcriptome Sequencing, De Novo Assembly, and Transcript Analysis

Sequencing of the venom gland transcriptome of the 20 specimens of *P. depilata* was generated from mean 51.6 million paired-end reads for each transcriptome library, with a minimum output per library of 40.4 million reads. Between 95 and 96% of the bases of the raw reads had a ≥Q30 quality score. Around 98% of the reads in all libraries passed the quality filters: remaining adapter removal, no ambiguous bases, ≥Q30, length ≥70 bases, no singletons (Supplementary Table S1). Raw sequences were deposited in the SRA database and can be accessed at NCBI BioProject PRJNA871796.

In order to perform a deeper quality control of the filtered reads of each library, individual de novo assemblies of each transcriptome library were performed, and the average contig size was calculated for each one. As can be seen in Supplementary Table S1, the minimum average contig size was 761 bases, with an average of 966 bases.

Since there is no reference genome for *P. depilata*, and with the goal to have a reference to map the RNA-seq reads, we generated a global transcriptome by merging the reads of at least one replicate of each group and assembling them with Trinity. With this strategy, we generated a global transcriptome that represented all the tested conditions as well as both sexes. The global transcriptome was the sum of 231,701,940 bases and was represented in a total of 216,190 contigs, with an average size of 1071 bases. The largest contig spanned 23,855 bases. No ambiguous (Ns) bases were present in the global transcriptome.

In RNA-seq experiments like this one, in which the tissues do not come from sterile material, and in which the microbiota of the spider can be present in the sample, it is recommended to classify the assembled contigs, in order to filter out contaminating assembled sequences. With this aim, we compared the contigs-assembled global transcriptome dataset with the NR NCBI protein database using the BLASTX tool, and then the results were classified using MEGAN software. We decided to do this analysis with the contigs obtained of the reads, due to the fact that longer nucleotide sequences, in this case contigs, might have a better performance in the taxonomic assignment since longer alignments will give higher alignment scores. MEGAN results showed that, although some contigs were assigned to bacteria, fungi, and metazoans, most of them were successfully assigned to Arachnida, in total 52,863 (Supplementary Figure S1). The contigs assigned to Arachnida were selected as the reference global transcriptome of *P. depilata* and used in the subsequent RNA-seq library mapping analysis and raw count measurements with htseq-count software.

### 2.2. Differential Expression

Differential gene expression analysis was performed using the DESeq2 package in the R environment. The output results of all tested groups are presented in Supplementary Table S2. The DESeq2 program printed results for 17,555 contigs. This result is expected in this type of program, since contigs with 0 counts in the mapping step are excluded from the analysis, and counts of repetitive DNA sequences are ignored in the output of the htseq-count software.

**Figure 1:**
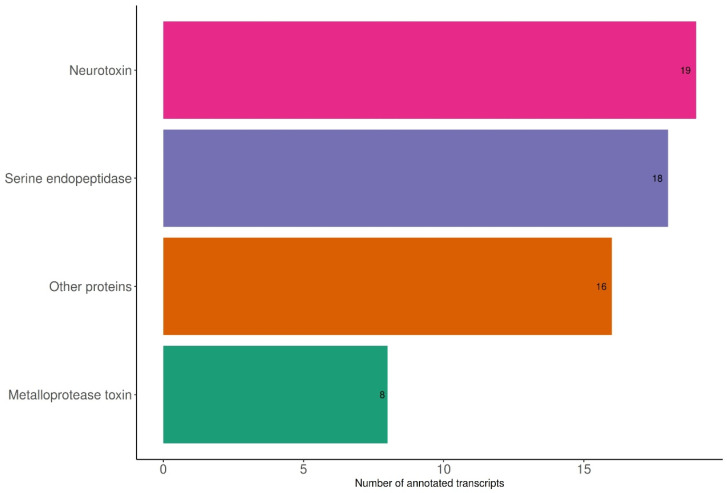
Classification of transcripts differentially expressed related to venom production in *Phoneutria depilata*.

The design including only sex as a factor produced a total of 488 differentially expressed transcripts (DETs) (Supplementary Table S2; DET total). From this list only 65 transcripts were related to venom production. They were classified as neurotoxins (36), serine endopeptidases (14), other proteins related to venom production (11; including acetylcholinesterases, hydrolases, and chitinase), metalloprotease toxins (3), and a venom potentiator (1). Also, we did not detect any differential expression of venom components of major importance, such as cytolytic compounds (Figure 1, Supplementary Table S2).

The design including only diet (~DIET) gave a very low number of DETs in each contrast (Mix~Vertebrate = 3, Mix~Invertebrate = 29, Vertebrate~Invertebrate = 6) (Supplementary Table S2), not providing enough support to draw any possible conclusion.

**Figure 2:**
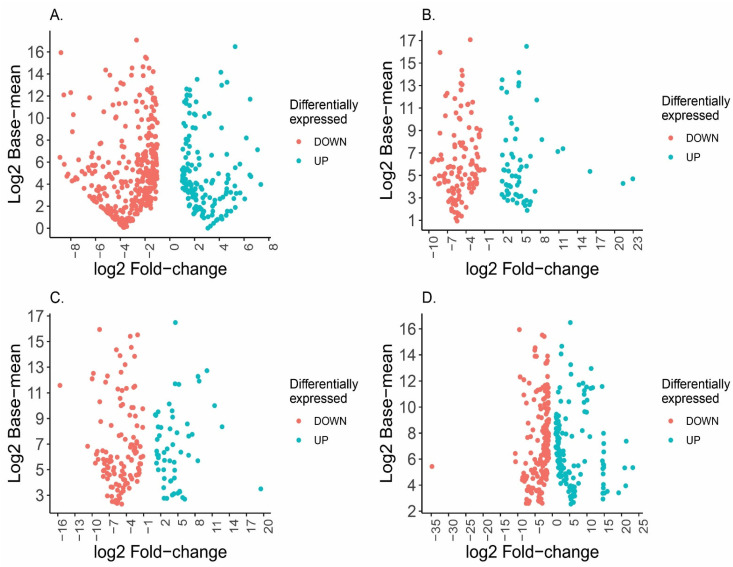
Volcano plot of analysis of transcripts significantly differentially expressed in *Phoneutria depilata*. Down transcripts differentially expressed are indicated as pink spots and up as the blue plot. (**A**) SEX_only, (**B**) Invertebrate diet, (**C**) Mixed diet, (**D**) Vertebrate diet.

When considering the design including the interaction between sex and diet, it was under a model of female versus male samples (independent and dependent of diet) in which it would be possible to identify the transcripts that were differentially expressed across the sexes in all the three different diets. The number of different DETs detected in the three treatments was 181 in the invertebrate-based diet, 174 in the mix-based diet, and 315 in the vertebrate-based diet (Supplementary Table S2). The number of differentially expressed transcripts in this model is lower than the ~SEX model, but in turn these transcripts have more extreme log2 fold change values (Figure 2). This phenomenon is possibly due to the fragmentation of the samples across the different groups, increasing the within-group variance and leading to an overestimation of the LFC values.

**Figure 3:**
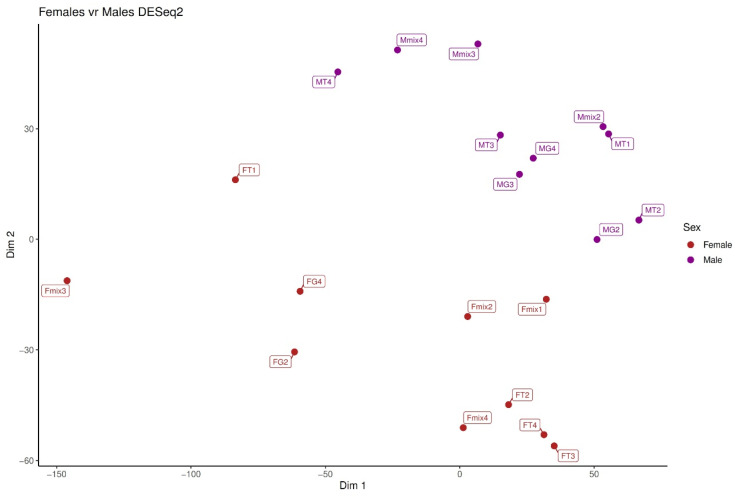
PCA analysis of transcripts significantly differentially expressed between males and females but across the three different diet types in *P. depilata*.

Figure 3 shows how 59 transcripts were differentially expressed between males and females but shared across the three different diet types. In all, 14 transcripts were shared across the vertebrate and the invertebrate diet; 23 were shared across the vertebrate and mixed diet, and finally 22 transcripts were uniquely shared across the invertebrate and mixed diet.

Our results show that the total number of transcripts differed between the ten males and ten females is of 5,309,740, with a means by sample of 265,487. However, the mean number of transcripts among females is 418,166 (Standard Deviation (SD) of 263,840) compared to a mean of 112,808 in males (SD 53,589). This is a significant difference between the numbers of transcripts expressed in the two sexes (*p* < 0.001). Likewise, when considering only diet as a factor, a slightly higher number of expressed transcripts is observed in the vertebrate diet group (299,081 ± 214,885) than in the mixed diet (246,929 ± 254,965) and invertebrate diet (316,913 ± 308,802). The number of differentially expressed transcripts per gene and per isoform in each sample normalized is summarized in the Supplementary Table S3.

The most expressed genes were the *Tx3-2 neurotoxin*, *Mu-Ctenitoxin-Pn1a*, and *cystine knot toxin*, with 48.4% (2,573,441/5,309,740), 19.6% (1,042,095/5,309,740), and 7.3% (392,079/5,309,740), respectively (Supplementary Table S3). Likewise, differences in the copy number of transcripts between males and females in these three genes were observed. For example, in *Tx3-2 neurotoxin*, the mean number of transcripts among females is 217,667 ± 199,775, compared to a mean of 39,676 ± 22,302 transcripts observed in males. These differences were significant (*p* = 0.004072). Similar behavior was observed in the *Mu-Ctenitoxin-Pn1a* and *cystine knot toxin* genes as well as in the other genes analyzed (see Supplementary Table S3).

## 3. Discussion

This work represents the first comparative venom gland transcriptome analysis conducted for *P. depilata* across three diets (vertebrate, mixed, and invertebrate) and the sexes. Our results indicate that there are no differences in DETs across the diets analyzed, but there are significant differences between the sexes, with 59 transcripts being differentially expressed. The mean number of transcripts in females is three times higher (418,166) than in males (112,808). The results show that although the same genes are expressed in both sexes, the venom glands of females express a greater number of components than those of males (see Supplementary Table S3).

On the other hand, we detected a high variation in gene expression at the intra-sexual level (an SD of 263,840 in females and an SD of 53,589 in males, respectively); which would imply that each individual has the capacity to regulate the quantity of venom components but not its quality. Although there were no significant differences in gene expression in the venom when diet was analyzed independently from sex, more transcripts were expressed in the invertebrate diet treatment (316,913) compared to the vertebrate (299,081) or mixed diet (246,929) (see Supplementary Table S3). However, the interaction between diet and sex provided some more insights about which transcripts might be differentially expressed across diet if the sex is taken into account.

Variation in animal venom toxicity and composition exists at both the inter- and intra-specific level, and several factors such as geographical location, venom synthesis rates, diet, and sexual dimorphism may contribute to this variability [20,31,33,50]. The difference in spider venom composition and/or potency between sexes has been shown across several spider families [9,31,32,33]. These studies show that venom composition varies in the presence of certain components in either sex or components that are common to both sexes but are expressed in different amounts [31,32,51]. For example, in the *Tetragnatha* species, a sexual dimorphism in venoms may function outside of prey capture or defense against predators and have particular potential for roles in intraspecific interactions, including sexual interactions [32]. The predator’s diet may also influence the composition of the venom. Wilder and Simpson [52] have shown that the diet of redback spiders (*Latrodectus hasselti*) can be composed of prey of high nutritional quality (e.g., fence skinks or locusts) and/or low-quality arthropod prey (e.g., beetles). However, this spider was more efficient at extracting nutrients (dry mass, lipid, and protein) from prey such as locusts and less efficient (extracting lipid, dry mass, and protein) from prey such as skinks and beetles. Nevertheless, this “feeding efficiency” (refers to how efficiently an animal utilizes its feed) is often associated with sexual dimorphism in spiders [32]. The differential expression of venom genes we observed between sexes in *P. depilata* could be correlated with a higher feeding efficiency in females than in males. The most evidence in favor of this hypothesis is provided by Sierra et al. [44] who detect intersexual differences in the trophic ecology of the species. Using the DNA metabarcoding technique, the authors found that females consumed fewer prey species (28.9%), compared to males (71.1%). This could be interpreted to mean that females consume fewer but higher quality prey than males. Females of *P. depilata* have the ability to include small vertebrates in their diet due to their larger size [43,44]. However, it is unclear if vertebrate prey is of higher quality than invertebrates.

Another point in support of the hypothesis of feeding efficiency in females of *P. depilata* is the differential expression of neurotoxins observed in this work (see Figure 2). Our data indicate that some of the major venom components differentially expressed in this spider species are the toxins *Tx3-2 neurotoxin*, *Mu-Ctenitoxin-Pn1a*, and *cystine knot toxin*. For example, *Tx3-2 neurotoxin* (48.4% of all expressed genes) is more than five times more expressed in females (217,667) than in males (39,676), with a similar pattern in the *Mu-Ctenitoxin-Pn1a* and *cystine knot toxin* genes. The *Tx3-2 neurotoxin* is not only detected in the venom of the genus *Phoneutria* but also in other spiders such as *Hololena curta* and *Agelenopsis aperta*. These toxins cause neurological symptoms in mice after intracerebroventricular injection [53].

Previous analyses have classified these three toxins as cysteine-rich peptide toxins that act on different ion channels. This type of toxin has been studied in other species of the genus such as *P. nigriventer* and is the main component of its venom [46,54]. These cysteine-rich peptide toxins have been shown to interfere with the sodium channels, generating depolarization in neurons in vitro, and in mice it induces excitatory symptoms and spastic paralysis [42,54,55]. Other toxins such as U2-ctenitoxin and U20-ctenitoxin have shown effects in vertebrates, such as almost instantaneous flaccid paralysis when applied to the cerebral ventricle of mice [54]. In the same way, other toxins such as Delta-ctenitoxin, which has been reported in the venom of *P. reidy*, also affect sodium channels but generate rapid general spastic paralysis and death when injected in mice at dose levels of less than 2 µg per mouse [54]. This potency demonstrates its relevance in the venom of females, whether in feeding on vertebrates or in defense against them.

Males express some neurotoxins in a greater amount than females, such as U5-ctenitoxin. This toxin can cause tail erection in mice and a slight reduction in mobility at a dose of only 1.40 µg per mouse [54]. Additionally, another protein overexpressed by males was Gamma-ctenitoxin, which has been shown to be highly toxic in the insects *Musca domestica*, *Acheta domesticus*, and *Periplaneta americana*, while in rats it only shows an anti-nociceptive effect [56]. This result is consistent with the varied diet of insects that males of *P. depilata* have [44]. In the same way, the venom of males has been shown to be more lethal to insects than the venom of females, even if it is administered in lower doses [43]. This is probably caused by the greater expression of genes that code for gamma-toxins and *U12-ctenotoxins*. Another possible explanation for the difference in expression between females and males is that to be efficient, the venom injected must have the same effects regardless of the amount. Similarly, if the role of defense is evaluated, and if the selective pressure is the same for both sexes, the lethality should also be the same.

## 4. Conclusions

This study shows differential gene expression by sex in *Phoneutria depilata*. Most of the differentially expressed genes are related to the production of neurotoxins, some of which have been shown to be very effective in vertebrate models, suggesting ecological implications. Our results support new evidence in favor of the biological basis of sexual dimorphism since the higher production of neurotoxins in females of this spider would be correlated to greater aggressiveness and defense against predators than males, a pattern also seen in some scorpions.

## 5. Materials and Methods

### 5.1. Collection and Differential Diet Experiment

**Figure 4:**
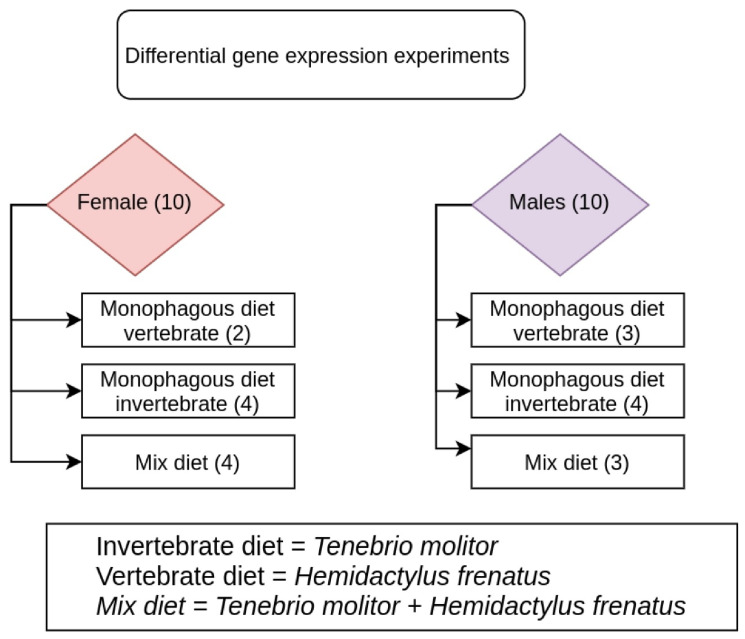
The methodological design of differential gene expression of the spider *Phoneutria depilata* (Araneae, Ctenidae) venom glands.

A total of 24 adult specimens of *P. depilata* were used for a differential diet experiment. All specimens were collected in the municipality of Oporapa-Huila (2°00′47.5″ N 76°00′02.6″ W) by night sampling on 24 September 2020 between 21:00 and 03:00 h and were transported to the biology laboratory of the University of Ibagué (Ibagué, Colombia). The experiment initially consisted of 12 females and 12 males, distributed in three different treatments (Female = 4, Male = 4 for each treatment): a vertebrate monophagous diet (*Hemidactylus frenatus*; Squamata, Gekkonidae), an invertebrate monophagous diet (*Tenebrio molitor*; Coleoptera, Tenebrionidae), and a mixed diet (*H. frenatus* + *T. molitor*). Each individual of *P. depilata* was fed ad libitum in an independent container under controlled laboratory conditions (24 °C and 75–80% RH) for six weeks, a timespan previously standardized by previous studies with arachnids [48]. Despite some concerns about the potential effect of distinct feeding procedures on venom transcripts under laboratory conditions, this issue remains to be tested and confirmed, since it is necessary to consider the presence and differential content analysis of other venom components and specifically the transcripts resulting from other experimental trials, e.g., overfeeding vs. dietary restriction. During the experiment, four specimens died (two females and two males), leaving a total of 20 specimens. The methodological design is summarized in Figure 4. After the six weeks of treatment, all spiders were sacrificed by freezing in liquid nitrogen. The extraction of the two venom glands for each individual was standardized previously, including some of the surrounding tissue of the glands in a careful process so as not to damage the venom glands. Venom glands were dissected in a sterile environment and macerated with a mortar in liquid nitrogen. The ground tissue was immediately stabilized with TRIzol reagent.

### 5.2. RNA Extraction, Library Construction, and Sequencing

Total RNA was extracted using TRIzol reagent following the manufacturer’s instructions (Ambion, Life Technologies, Carlsbad, CA, USA). Total RNA integrity was assessed using an Agilent 2100 Bioanalyzer. The mRNA integrity was evaluated in a 2100 Bioanalyzer, picochip series (Agilent Technologies, Waldbronn, Germany). The mRNA library preparation and NGS sequencing were outsourced to Macrogen (Seoul, Republic of Korea). The mRNA library was generated using the TruSeq RNA Sample Prep Kit protocol (Illumina, San Diego, CA, USA). The NGS sequencing was performed on the Illumina NovaSeq™ 6000 platform (Illumina, San Diego, CA, USA), sequencing 100 bp paired-end reads.

### 5.3. De Novo Transcriptome Assembly

Raw reads were filtered using Cutadapt v3.5 software with options -q30-m70-max-n0. Remaining sequences of Truseq adapters were removed as well as low quality reads. The Cutadapt quality threshold was set to Q30, and reads shorter than 70 bases were discarded. The de novo transcriptome assembly was performed using the assembly pipeline Trinity version 2.13.2 [57]. The descriptive statistics of the Trinity assembly were calculated with a custom Python script written at the CNSG lab.

### 5.4. Clustering and Functional Annotation of Transcripts

MEGAN [58] was used to filter out contaminating transcriptome data considering all invertebrate contigs as sequences of *P. depilata*. Additionally, a BLASTx alignment (E-value cut-off of 10^−30^) was performed with protein databases obtained with the R package rentrez version 4.0.3 [59] and KEGG database. The assignment of KEGG pathways to the transcripts was carried out using KEGG Automatic Annotation Server (KAAS; https://www.genome.jp/kegg/kaas) accessed on 1 December 2022 using the bi-directional best-hit method.

### 5.5. Differential Expression Analysis

Raw counts of the Trinity assemblies were obtained with htseq-count software [60], and subsequently we used the DESeq2 v.1.36.0 software [61] to identify DETs following three different designs. Differential expression analysis is generally performed in a univariate (or unifactorial) way, meaning that only one factor is being analyzed (either diet or sex as in the first two models). Our analysis aims to understand how two different factors may affect the differential expression of toxin genes. The interaction term is added to the design formula, in order to test, for example, if the log2 fold change value attributable to a given condition is different based on another factor, for example, if the condition DIET differs across SEXES. If males and females do not have a different response to the diet type, there is no need to add the interaction terms because sex affects in the same direction in the response to diet. If we want to test whether one sex responds in one way and the other sex in the opposite, we need to include the interaction term across sex and diet in our models.

From the output of the function DESeq, a list of transcripts having a log2FoldChange (LFC) ≠ 0 was retrieved. From this list, all the transcripts accounting for less than 10 reads in total were filtered out. All transcripts were normalized using the DESeq2 tool. After this first filter, only transcripts having an FDR of <0.05 and a log2FoldChange less than −1 and greater than +1 were kept. To assess if any outlier was affecting the LFC, the normalized counts per DET after all the filtering were plotted. Each DET accounting for a total of outliers ≤half of the group size was considered as a false positive and discarded from the final list of DETs.

### 5.6. Categorization of Transcripts

The annotated and differentially expressed transcripts were categorized by manually checking the BLASTx matches in the UniProtKB/Swiss-Prot and PDB databases [62,63], considering whether they had been previously reported as related to the production of venom including neurotoxins, cytotoxins, chitinases, and proteases.

## Data Availability

The data presented in this study are available in Supplementary Materials and Genbank database.

## Supplementary Materials

The following supporting information can be downloaded at: https://www.mdpi.com/article/10.3390/toxins15070429/s1. Figure S1: Classification of transcripts for 20 transcriptomes of *P. depilata*. Assessment performed using BLASTX and MEGAN. Table S1: RNA extraction and pre-processing values for 20 transcriptomes of *P. depilata* Table S2: Classification of differentially expressed transcripts detected in *P. depilata*. Table S3: Number of DETs between the ten males and ten females by sample.

## Abbreviations

DESeq	Differential gene expression analysis
DETs	Differentially expressed transcripts
DIET	Design including only diet
NCBI	National Center for Biotechnology Information
