# Supplementary figures and images for: Intersexual Differences in the Gene Expression of Phoneutria depilata (Araneae, Ctenidae) Toxins Revealed by Venom Gland Transcriptome Analyses
Source: Toxins (Basel). 2023 Jun 30;15(7):429. doi: 10.3390/toxins15070429 (PMC10467060; doi:10.3390/toxins15070429)

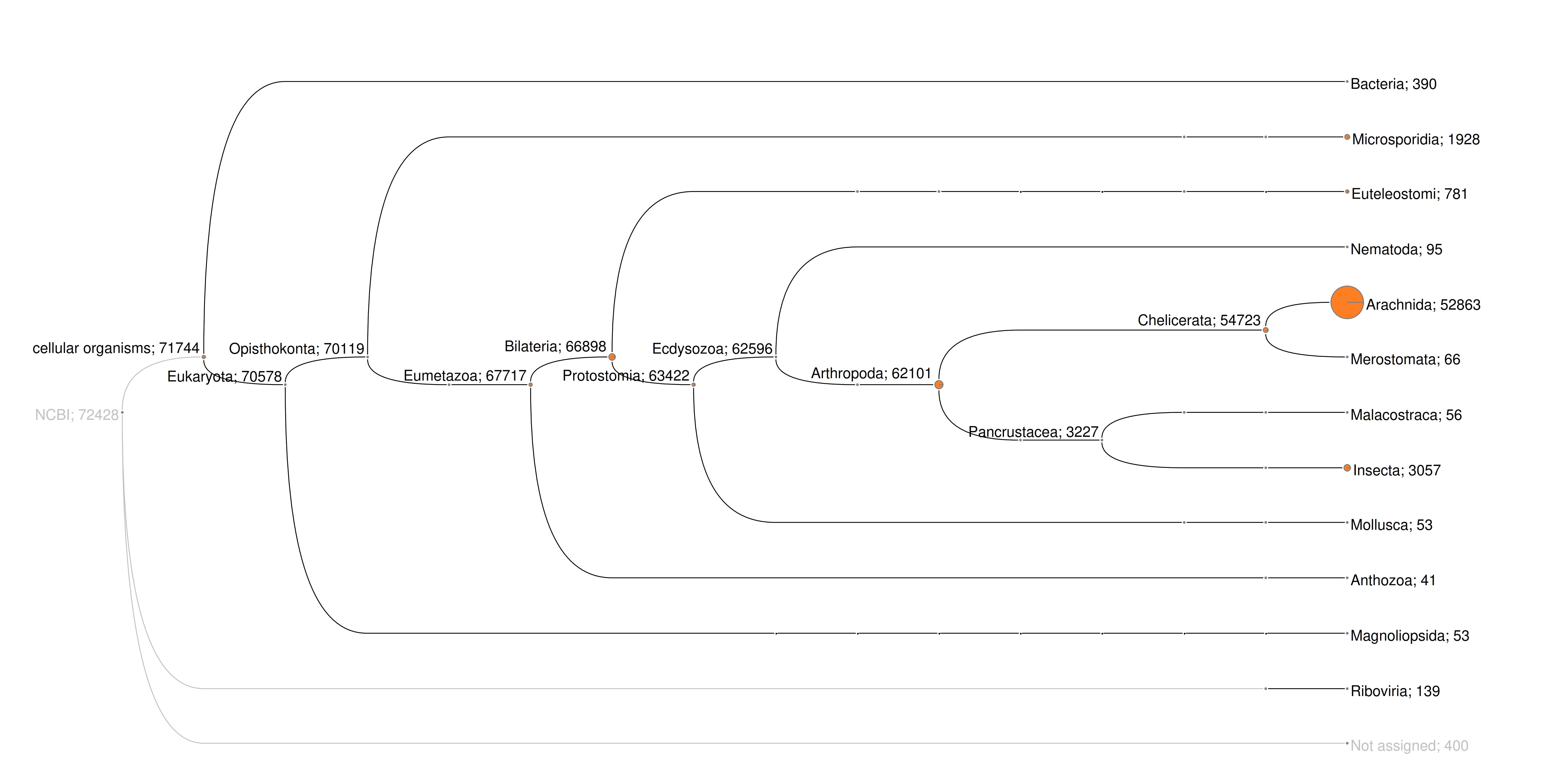

Supplement: Supplementary file 1 [file toxins-15-00429-s001.zip › Supplementary Figure S1.jpg]
